# Dynamic multilevel spiral phase plate generator

**DOI:** 10.1038/s41598-018-34041-2

**Published:** 2018-10-25

**Authors:** M. Caño-García, X. Quintana, J. M. Otón, M. A. Geday

**Affiliations:** CEMDATIC, E.T.S.I Telecomunicación, Universidad Politécnica de Madrid, Av. Complutense 30, 28040 Madrid, Spain

## Abstract

The design and characterisation of a reconfigurable multi-level spiral phase plate is shown. The device is based on a pie-shape liquid-crystal structure with 24 slices driven by custom electronics that allow independent excitation control of each electrode. The electrooptical cell was manufactured using maskless laser ablation lithography and has shown an unprecedented high fill factor. The topological charge can be dynamically changed between 1, 2, 3, 4, 6, 8 and 12. The device has been calibrated and characterised at 632.8 nm but can be employed at any wavelength in the visible and near infrared spectrum, just modifying the driving parameters of the electrodes. The experimental results have been compared to predictions derived from simulations. An excellent correspondence between theoretical and experimental result has been found in all cases.

## Introduction

Optical vortices were first mentioned in the pioneering work of Nye and Berry^1^ in the 70’s and the first reports on generation of optical vortices were published two decades later^2,3^. Since then interest into vortices has been growing with the increasing number of applications as diverse as cooling and trapping of droplets^4^, neutral atoms and Bose-Einstein condensates^5,6^, manipulation of microscopic particles^7^, optical communications^8^, solar corona^9^, exoplanet observation^10^ and stimulated emission depletion microscopy^11^.

The term *optical vortex* is used to describe a singular point of zero intensity inside an optical field or a paraxial light beam containing such a singular point. In this work the term will refer to the latter definition.

**Figure 1:**
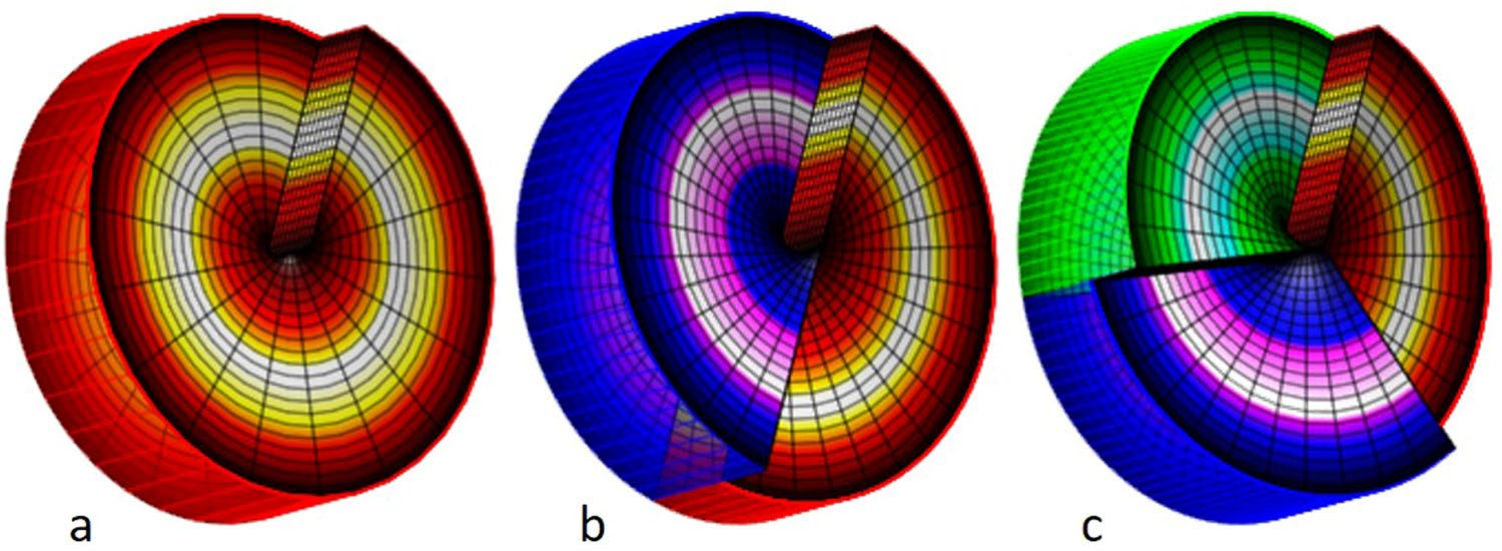
Examples of SPPs that convert planar incident light into optical vortices with a topological charge of 1, 2 and 3 respectively. The gradual increase and discontinuity jumps in each of the phase surfaces corresponds to a phase difference of 2π.

Optical vortices may be produced in various manners, but the easiest way to appreciate vortices is to consider the simplest vortex producing device, *the spiral phase plate* (SPP), which introduces an azimuthally varying retardation in a wavefront as illustrated in Fig. 1.

**Figure 2:**
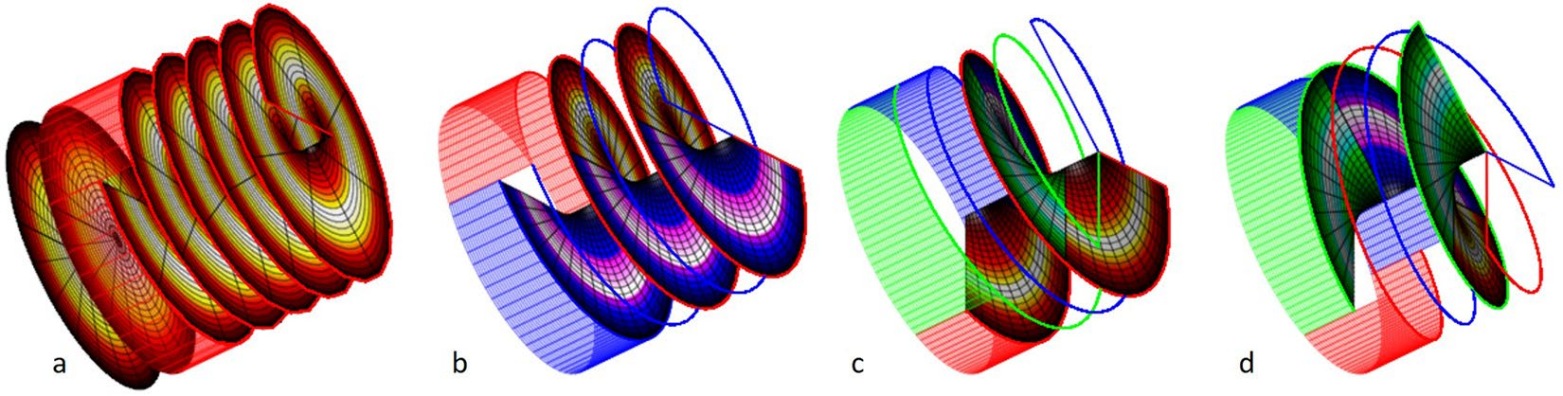
The single, double and two triple helices (vortices), resulting from an incident planar wavefront (only indicated in **a**). In (**b**–**d**) only one of the helical constant phase surfaces is fully drawn, the remaining are indicated by a line only. The vortex in d has the opposite hand to the others.

Each of these phase plates convert a planar wavefront into an integer number of entwined helices, *an optical vortex* (Fig. 2).

An optical vortex is characterised by its *orbital angular momentum* or *topological helical charge*, *l*, which may take any integer non-zero number, and is equivalent to the number of entwined helices describing the wavefront. Positive and negative values of *l* describe the handedness of the helices: righthanded vortices are conventionally assigned to positive helical charge.

The central axis of the vortex phase distribution contains all phase values ranging from 0 to 2π·*l*, giving rise to a singular point of destructive interference and the formation of a ring-shaped beam in the far-field with a radius proportional to *l*^12^. The ideal SPP introduces a continuously varying azimuthal phase delay, apart from at the *l* number of discrete angles where the relative phase delay corresponds to a full wavelength *i.e*. where 2π phase wrapping occurs.

Due to fabrication limitations rather than a continuously varying phase delay solid state SPPs have a finite number of discrete phase delay levels^13,14^.

Alternatively, high quality vortex beams with a fixed topological charge may also be generated by sub-wavelength gratings^15^, annular gratings^16^, meta-materials^17^ or by using computer generated holograms^18^.

The liquid crystal (LC) phase may be organised in such a way that it generates optical vortices. Since LC is, generally, an anisotropic phase, LC devices require the incoming light to have a given polarisation state, typically, linearly or circularly polarised. Selectively orienting the molecules in structures, such localized umbilical defects^19^ or droplets^20^ in the LC phase or by using *q-plates*^21,22^ may result in optical vortices, with a certain degree of reconfigurability. However, more commonly electrically addressed spatial light modulators (SLMs) are used^23,24^.

SLMs employing nematic LCs are characterised by a preferred alignment direction, which together with the applied electrical field vector, parallel to the incident light beam, defines the switching plane of the LC. The switching state of the LC defines the refractive index for the light polarised parallel to the switching plane. In SLMs the LC is selectively switched spatially and thus generating a specific phase retardation pattern.

Conventional SLMs are generally hampered by a pixelated structure that generates aliasing and reduces the fill factor of the device leading to a loss of efficiency. Less generic electro-optical reconfigurable LC multi-level SPP devices for generating optical vortex based on discrete^25^ or interconnected^26–28^ electrodes, with reduced interpixel space have been demonstrated.

This work reports the design, simulation and manufacturing of a reconfigurable LC multi-level SPP, with minimal interpixel-space and maximal direct control of the individual switching sectors. The LC cell is based on the previously reported device^25^ with several novelties: the electrodes pattern is created using a maskless laser ablation^29,30^, resulting in a very high fill factor of electrodes; the manufactured device consist in 24 pie slices sections which is twice as many as any commercially available SPP; a custom made cost-efficient, pulse-width modulation driver was developed and manufactured for device driving; finally the manufacturing protocol makes it possible to increase the number of sections arbitrarily, without limitations in neither the fabrication process nor in the driver.

## Methods

### Manufacturing

An LC cell consists of two opposing transparent ITO electrodes treated with an alignment agent, separated by spacers. The gap between the electrodes filled with an LC and sealed.

A CAD program was used to design the electrode layout. A 50 × 50 mm^2^ ITO covered 1.1 mm thick glass slide was used for the patterned bottom electrode and single electrode of 30 × 20 mm^2^ ITO glass slide was used as counter electrode

**Figure 3:**
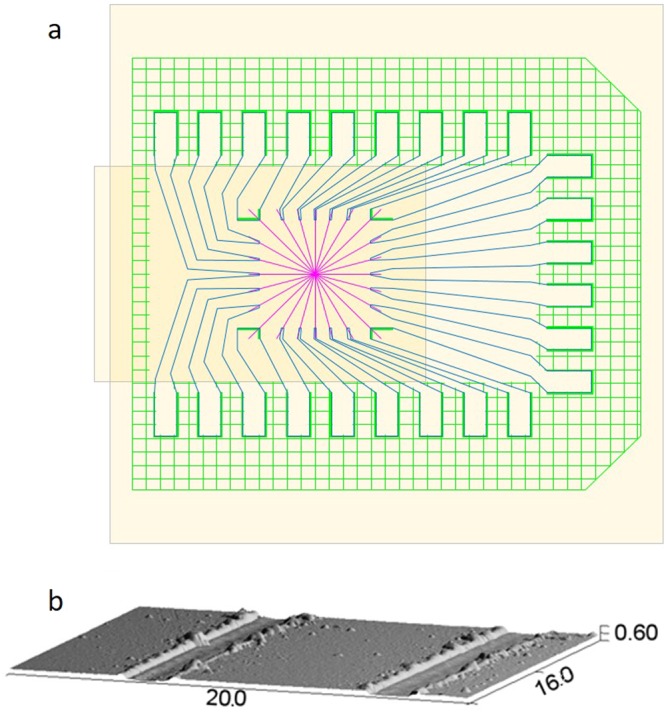
The LC electrodes. (**a**) The manufactured electrode design. The patterned base glass (50 × 50 mm^2^), and the single electrode is presented in transparent yellow. The active area (10 × 10 mm^2^) is presented in purple, the fan-out (blue) connect the electrodes with the active area. In green, several cuts to ensure the isolation between pads and to facilitate the assembly. (**b**) Example of the laser ablation leading to electrode separations of approximately 2 µm. The imaged area measures 20 × 16 × 0.6 µm^3^.

The active area is 10 × 10 mm^2^. The active electrode pattern consists of 24 pie slices. Each pie slice is connected to a contact pad measuring 2 × 4 mm^2^ (Fig. 3a).

The ITO was engraved by CAD controlled ablation system (Lasing S.A., Madrid, ES) fitted with a 300 mW, 349 nm laser (Explorer Laser Spectrum Physics, Mountain View, US), using a back-scribing approach, writing through the glass^30^. The resulting ITO electrode separations were in the order of 1.5–2.0 µm (Fig. 3b).

The minimal electrode separation is important particularly in the centre of the active area, where all the cutting lines meet. The design was made so that different cutting parameters could be employed in different sectors.

Cells were mounted using rubbed polyimide as alignment layer and MDA-98–1600 as LC. The polyimide layer induces a homogenous alignment of the LC molecules parallel to the cell surface in the direction of the rubbing. The LC a is positive nematic, which will tend to align parallel to any applied electrical field. The degree of switching depends on the torque applied by the electrical field and the strength of the polyimide anchoring. The stronger the field applied, the more the molecules will tend to orient perpendicularly to the cell surface; once the field is removed the LC will relax back into the state dictated by the alignment layer.

**Figure 4:**
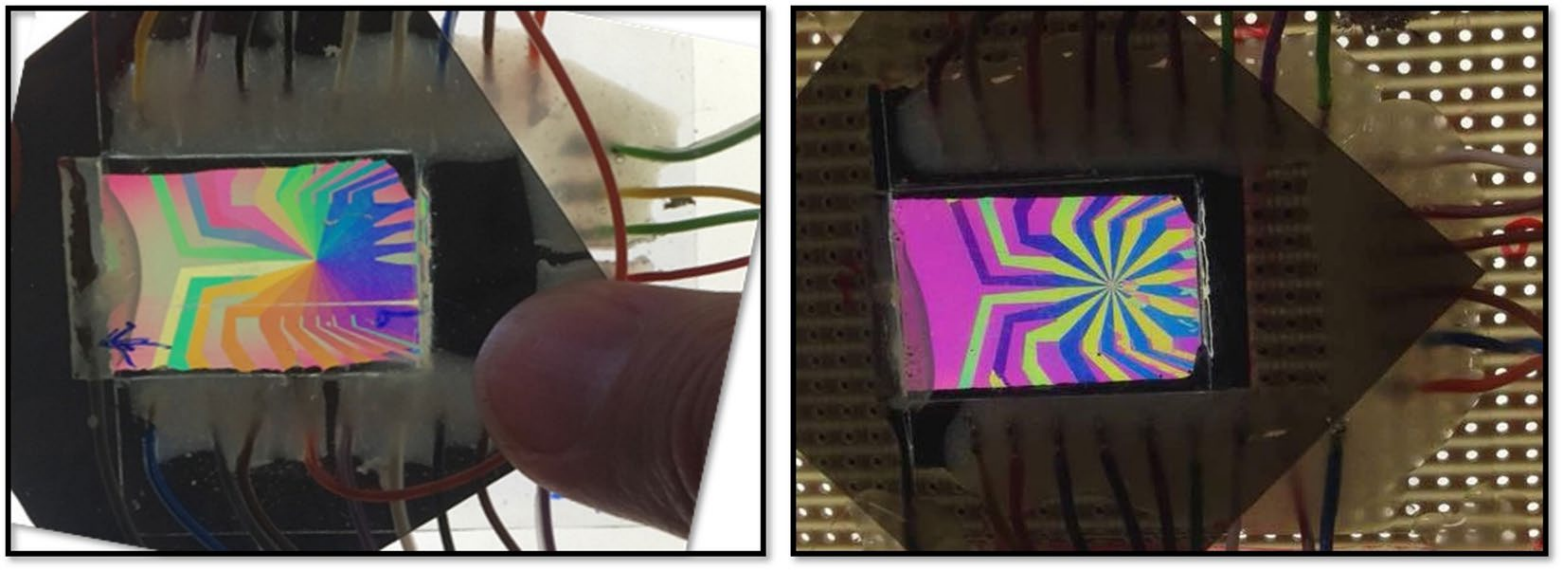
Examples of the reconfigurable SPP under crossed polarizers with different voltage applied (different colours represent different phase delay).

The cell thickness (*d*) was ensured using 9 µm wide silicon spheres as spacers in the sealing gasket. The cells were filled by capillarity action. After the LC filling, each cell was wired and electrically tested. The cells were connected to a multichannel voltage generator, described below, and were assessed in white light between crossed polarizers to check the quality of the sample (Fig. 4).

**Figure 5:**
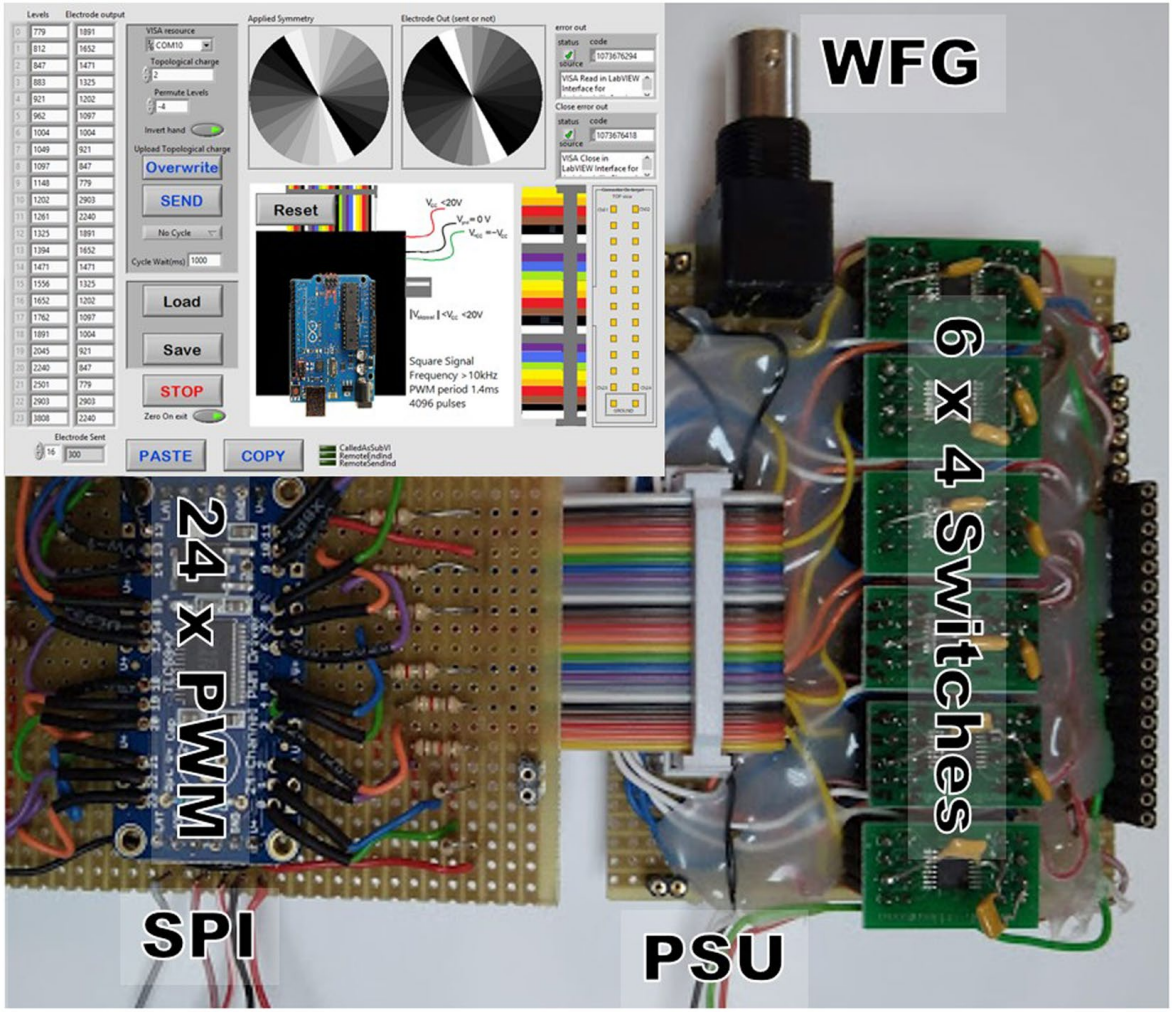
The basic components of the device driver. An array of 24 × 12 bits is sent to the 24 PWM via the SPI interface. Each of the PWM signals is pulled up by 1.8 kΩ resistor to make the output signal transistor-transistor-logic (TTL) compatible. Six four channel ADG5412 switches convert the TTL-PWM signals to effective rms voltages (0 to 6 V) for each of the 24 LC signals, by connecting to the 6 V, 10 kHz signal supplied by the waveform generator (WFG). The individual LC electrode is connected to the LC signals, and a 10 kΩ pull down resistor. The user interface is included in the insert.

A driver to control the vortex generator was manufactured. An Arduino UNO clone (Funduino) was employed as a USB-SPI interface for transferring a 24 × 12 bit array to a 24-channel 12-bit pulse-width-modulation (PWM) LED driver (*TLC5947, Texas Instruments* implemented in the *Adafruit 1429 board*). The 24 output channels of this driver were used as control inputs in six four-channel high speed analogue switches (*ADG5412 SPST, Analog Devices Inc*.) connecting, or not, the individual SPP electrodes to a 6 V, 10 kHz square waveform signal generated by an external arbitrary waveform generator (Stanford Instruments DS345) (Fig. 5). The refresh rate of the LED driver was measured to be 1.4 ms, which is far shorter than the relaxation time of a 9 µm thick liquid crystal cell.

**Figure 6:**
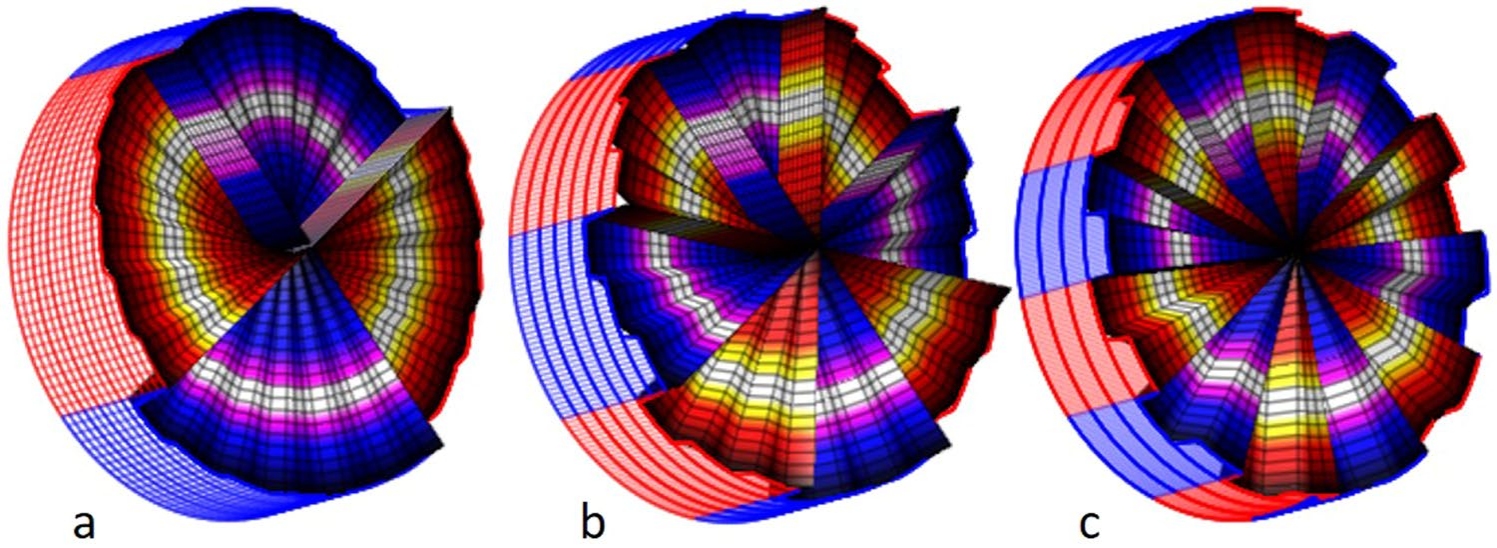
Examples of SPPs that are generated with 24 sectors electrode. The drawn SPPs have topological charges of 4, 8 and 12 respectively. Alternating helical wavefront components are coloured in blue and red colours.

The electronic driver, and the user interface allows for the generation of the desired multilevel discrete SPP profiles needed for the generation of the vortices (Fig. 6).

### Characterization

To establish the relationship between applied PWM duty cycle and phase retardation, the device was situated with the alignment direction (the switching plane) at 45° between two crossed polarizers, at 0° and 90° respectively, and illuminated by a He-Ne laser with a wavelength $(\lambda )$ of 632.8 nm. In this setup the intensity varies with the phase difference $(\delta )$ caused by the plano birefringence $({n}_{eff}-{n}_{o})$. of the birefringent LC of a thickness *d*.

$$I\propto si{n}^{2}\frac{1}{2}\delta =si{n}^{2}[\frac{1}{2}\frac{2\pi }{\lambda }({n}_{eff}-{n}_{o})d]$$

derived, employing Jones matrix formalism (see for instance Caputo *et al*.^31^). In this study, both camera dark current, and polariser imperfections have been ignored, since the resulting intensity variation is normalised. To ensure a CCD gamma factor of 1, only the green pixels were employed in the intensity calculations.

A digital camera (Nikon D500) fitted with a macro-lens was used for the light intensity reading. Raw image data for each separate colour pixel on the CMOS were captured.

In order to illuminate most of the device, the laser beam was expanded to 8 mm diameter using a beam expander.

Four centric regions of the device were chosen arbitrarily to be characterized, each region covering approximately 1/10 of the device. One common excitation signal was applied to the whole device during the characterization, and the intensity readings of each region were captured and averaged for each of the colour pixels separately.

**Figure 7:**
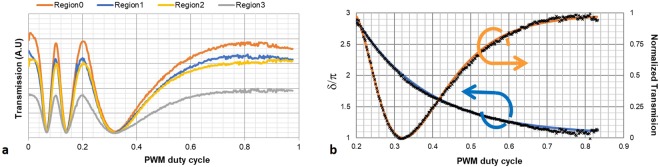
Cell calibration. (**a**) Transmission data for the green pixels in the four random regions as function of the PWM duty cycles. δ(∞) = 0 making it trivial to deduce δ from the measured intensity values. (**b**) The retardation (δ/π) with the fitted values (blue), and the normalized measured intensity with the fitted intensity (orange) as functions of the PWM duty cycle.

During the calibration the illumination over the sample was not perfectly uniform (Fig. 7a), however, this does not affect data acquisition, since only relative intensity variations are relevant.

The birefringence evolution as a function of the PWM duty cycle (*dc*) was found to be well described by a function $\delta =A{e}^{-B\cdot dc}+C\,\,$over a full 2π range $(\delta \in [3\pi ;\pi ])$. The agreement between the fit (A = 17.0, B = −5.38 and C = 0.15) and the experimental data is shown in Fig. 7b.

The fitted curve was employed to calculate the PWM for each retardation level used in the different topologies.

## Results

**Figure 8:**
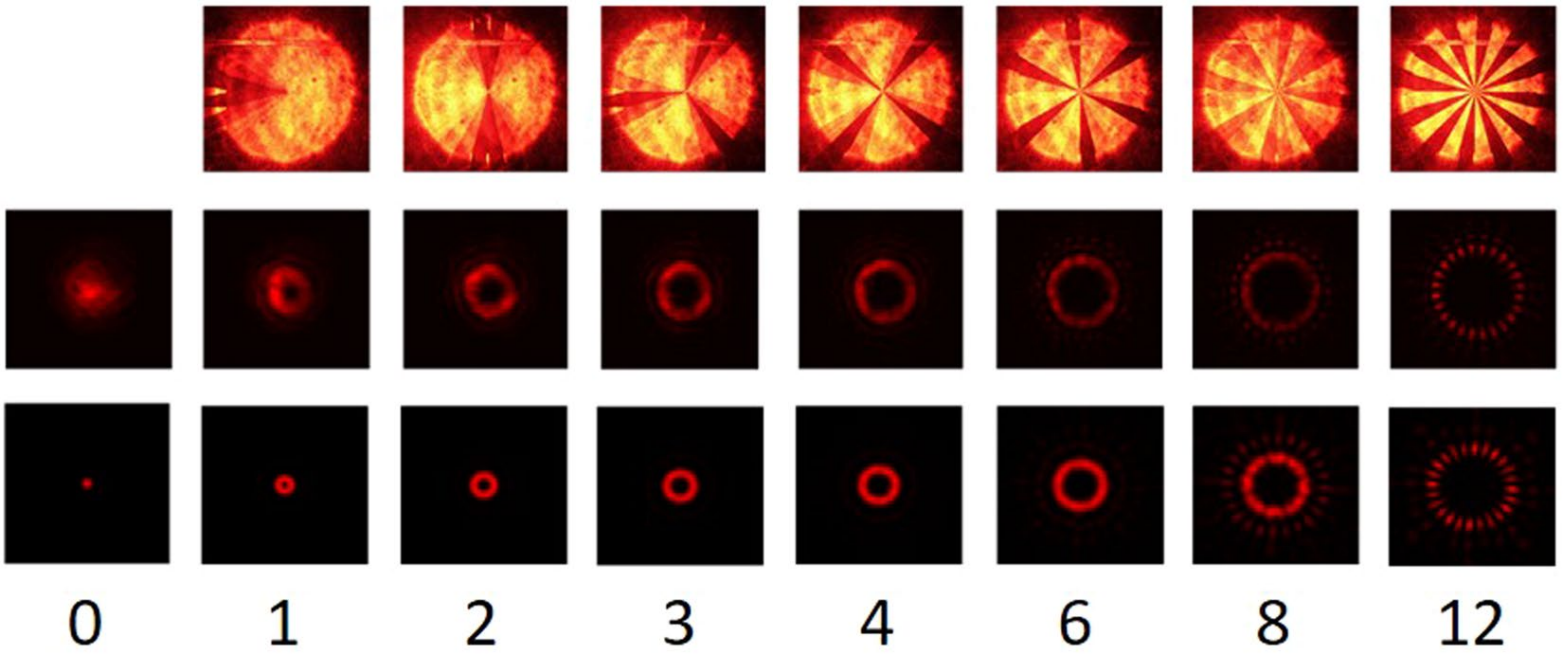
Light transmission. Top row shows the phase delay induced by sectors of the SPP by imaging the intensity transmission between crossed polarizers for the different topological charges [0, 1, 2, 3, 4, 6, 8 and 12]. In this row the width of the expanded laser beam was approximately 8 mm. The middle row shows the laser beam power distribution at 400 mm after the SPP. The width of the naked laser beam (leftmost image) was approximately 1.7 mm. The bottom row shows the simulated intensity reading of the light path for the corresponding topographies.

The 24 slices of the SPP allow for seven different topological charges [1, 2, 3, 4, 6, 8 and 12], employing 24 different excitation levels, deduced from the preceding calibration. The excitation of the different topologies was first imaged between crossed polarisers at 45° to the switching plane as employed for the calibration (Fig. 8 top row). The darkest areas correspond to minimum $\sin \,\frac{{\delta }}{2}$, *i.e*. where the introduced phase difference $\delta \approx 2\pi$, and the brightest areas correspond to $\delta \approx \pi$ or $\delta \approx 3\pi$.

Subsequently the second polariser was removed, and the SPP was placed such that the switching plane was aligned with the impinging polarization. The beam expander and the macro objective were removed, and the naked light beam originating in a conventional HeNe laser tube, with no focusing elements, was shone directly onto the camera CMOS chip.

The resulting intensity distributions can be seen in Fig. 8 (middle row). The zero-power density, singularity in the centre of the beam with a radius proportional to the topological charge, is clearly visible illustrating the expected functioning of the device. No significant power variations were detected when changing the sign of the topology or rotating the SPP profile.

All the camera and laser parameters were kept constant during the capture of the switching pattern and the diffraction patterns but changed in between the two sets of data. Thus, the intensity reading from image to image is directly comparable horizontally, but not vertically.

The diffraction pattern showed not only the principal diffraction ring, but also a weaker higher order diffraction ring (most visible in the image of the diffraction pattern of topology 8 (Fig. 8 middle row). This unexpected pin-hole like diffraction pattern motivated a comparison of the experimental results with a simulation of the setup.

A simple train of optical elements were cascaded mathematically to reach a description similar to the one proposed by others^28,32^: a Fourier transform of a gaussian power distribution of the initial laser, is filtered by a circular aperture and the SSP before being transformed back by an inverse Fourier transform into the power distribution depicted in the last row in Fig. 8. The simulation showed that the PIN-hole pattern was intrinsic to optical setup, and not a consequence of imperfections in the manufactured SPP. The major divergence between the measured and the simulated diffraction pattern is seen in the approximation to the initial gaussian distribution of the incident laser, and the actual measured power distribution.

The first row of Fig. 8 shows that imperfections were present in the presented SSP especially in the periphery of the device. The imperfections in the upper part of the device are due to a damaged alignment layer, probably caused by the manual handling of the device. The visible imperfection is approximately 5 mm from the device centre, which means that it does not affect the employed laser which, as seen in the first photo of the second row, has a 1.7 mm waist. In this latter image, it can be seen that the impinging light laser beam can only loosely be described as a gaussian beam.

Nonetheless, visually high contrast ratios are achieved in the images of the diffraction rings for all the topologies. The reason is that all the spatial intensity variation in the SSP and in the laser source is averaged out over the whole diffraction pattern. Simulations introducing random noise in both the laser intensity profile, as well as transmission and phase variation in the SSP, resulted in a reduction of the contrast of the diffraction patterns, in a direct proportion to the area, and intensity affected.

**Figure 9:**
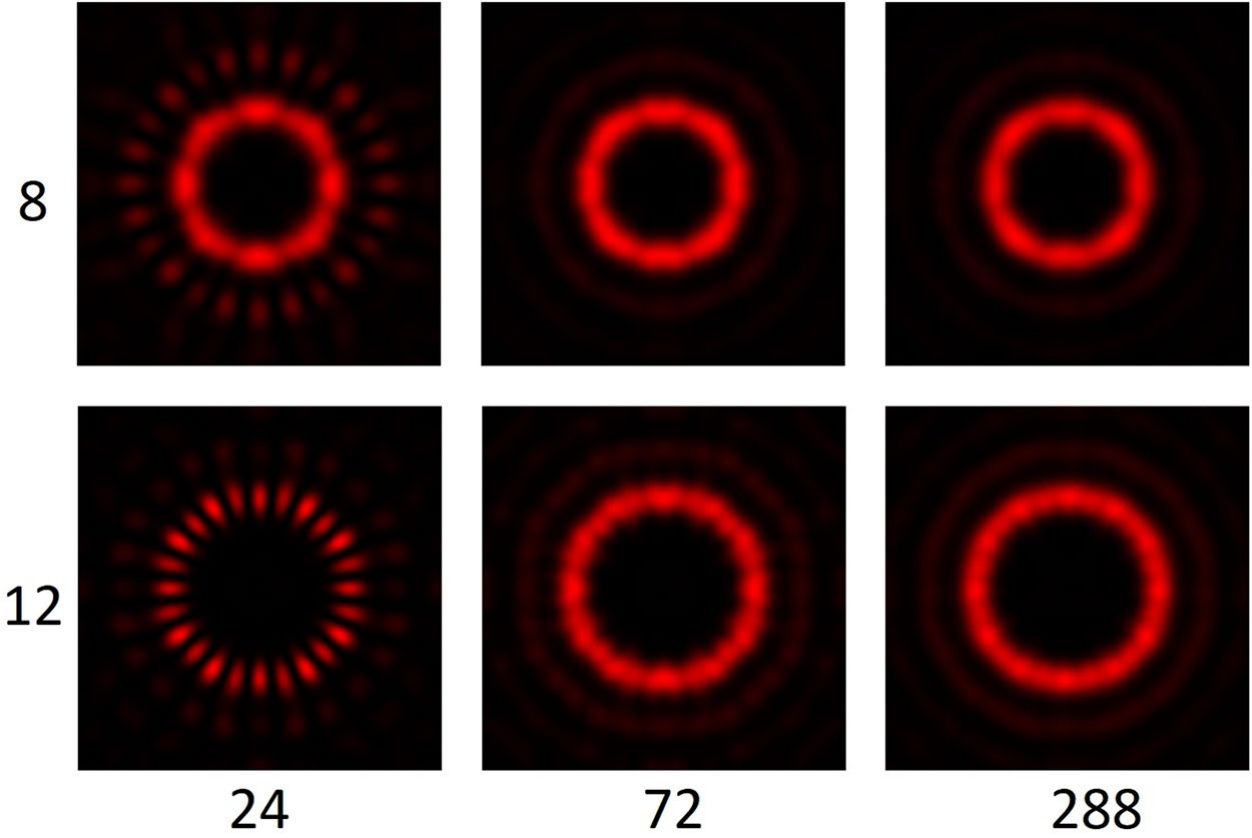
Simulated intensity reading of the light path for the topographies 8 and 12 in SSPs with 24, 72 and 288 pie slices. It may be appreciated that once the ratio between the number of slices and the topology exceeds 6, only marginal change in the ring neatness may be seen. The intensity simulations have been individually normalised, unlike in Fig. 8, where global normalisation was applied.

The simulation model allowed for evaluation of more complex devices. The neatness of diffraction ring depends on the number of pie slices that makes up the SSP. The closer to an infinite number of slices in each 0–2π period, i.e. a continuous variation, the neater the generation of the diffraction rings, as can be appreciated in the simulations shown in Fig. 9.

## Conclusions

A 24 pie-slice reconfigurable SPP with a custom-built driver has been manufactured and successfully tested. The electrodes were obtained using laser ablation. The device represents, to the knowledge of the authors, the SSP device with the highest degree of programming freedom (full independent switching control over each of the 24 electrodes), and the highest fill factor, with less than a ¼ mm^2^ (12 cuts each 10 mm long and 2 µm wide) of interpixel space in a 10 mm diameter (78 mm^2^) active area.

The device was designed to deliver retardations far larger than a full wavelength at the wavelength used in the characterization (632.8 nm). Consequently, the same device potentially could be employed NIR, with the same electronic driver and controlling software.
